# Salt-tolerance induced by leaf spraying with H_2_O_2_ in sunflower is related to the ion homeostasis balance and reduction of oxidative damage

**DOI:** 10.1016/j.heliyon.2020.e05008

**Published:** 2020-09-21

**Authors:** Petterson Costa Conceição Silva, André Dias de Azevedo Neto, Hans Raj Gheyi, Rogério Ferreira Ribas, Caroline Rastely dos Reis Silva, Alide Mitsue Watanabe Cova

**Affiliations:** a Centro de Ciências Agrárias Ambientais e Biológicas, Universidade Federal do Recôncavo da Bahia, Cruz das Almas, 44380-000, BA, Brazil; b Centro de Ciências Exatas e Tecnológicas, Universidade Federal do Recôncavo da Bahia, Cruz das Almas, 44380-000, BA, Brazil; c Centro de Pesquisas e Análises Clínicas, Cruz das Almas, 44380-000, BA, Brazil

**Keywords:** *Helianthus annuus* L., H_2_O_2_, Salinity, Oxidative stress, Physiological parameters, Cross-talk, Agricultural water management, Crop biomass, Crop production, Plant growth, Plant physiology

## Abstract

Salinity is still one of the main factors that limit the growth and production of crops. However, currently, hydrogen peroxide (H_2_O_2_) priming has become a promising technique to alleviate the deleterious effects caused by salt. Therefore, this study aimed to test different leaf spraying strategies with H_2_O_2_ for acclimation of sunflower plants to salt stress, identifying the main physiological and biochemical changes involved in this process. The experiment was conducted in a completely randomized design, with four replications. Initially, four concentrations of H_2_O_2_ were tested (0.1; 1; 10 and 100 mM) associated with different applications: 1AP - one application (48 h before exposure to NaCl); 2AP - two applications (1AP + one application 7 days after exposure to NaCl) and 3AP - three applications (2AP + one application 14 days after exposure to NaCl), besides this two reference treatments were also added: control (absence of NaCl and absence of H_2_O_2_) and salt control (presence of 100 mM of NaCl and absence of H_2_O_2_). The experiment was conducted in hydroponic system containing Furlani's nutrient solution. Salt stress reduced the growth of sunflower plants, however, the H_2_O_2_ priming through leaf spraying was able to reduce the deleterious effects caused by salt, especially in the 1 mM H_2_O_2_ treatment with one application. H_2_O_2_ acts as a metabolic signal assisting in the maintenance of ionic and redox homeostasis, and consequently increasing the tolerance of plants to salt stress.

## 1 Introduction

Salinity is considered one of the main problems encountered in agriculture worldwide. The effects of salinity are more conspicuous in arid and semi-arid regions, because limited rainfall, high evapotranspiration, high temperatures associated with inadequate water and soil management enhance the negative effects of salt stress and directly impact in the yields of crops (Katuri et al., 2019; Rodrigues et al., 2020).

The excess of Na^+^ and Cl^−^ ions in the root zone can alter the osmotic, ionic and nutritional homeostasis of plants (Cai and Gao, 2020). These changes can lead to reduced growth and affect several physiological mechanisms. Under these conditions, both photochemical and biochemical phases of photosynthesis can be negatively affected (Melo et al., 2020). Besides, salinity can increase lipid peroxidation, and consequently reduce the integrity of cell membranes, besides it causes an imbalance between the production and the scavenging of the reactive oxygen species (ROS) (Khan et al., 2019). This imbalance (oxidative stress situations) can cause cell damage, since ROS are very powerful oxidizers and can react with almost all components of living cells, producing severe damage to lipids, proteins and nucleic acids (Khan et al., 2019).

Therefore, understanding the mechanisms of plant tolerance to high concentrations of NaCl in soils can help improve yield and production in saline lands. Several studies using markers have been carried out in an attempt to improve the tolerance of crops to salt through conventional breeding programs, however this technique is too complex and expensive (Hoang et al., 2016).

As an alternative, plants can be prepared for future stress by priming. This technique, also known as sensitization or hardening, increases the plant tolerance to different types of stress. With the use of this technique, plants activate several protection mechanisms by different signaling pathway (Savvides et al., 2016).

Chemical priming is an emerging field in crop management under stress conditions. Plants treated with certain chemical agents (natural or synthetic) show increased tolerance when exposed to subsequent stresses (e.g., salinity, drought, heat, heavy metals) (Hossain et al., 2015; Niu and Liao, 2016; Savvides et al., 2016).

Among the several chemical agents used in this technique, is hydrogen peroxide (H_2_O_2_). Due to its electrochemical characteristics and small size, H_2_O_2_ can cross membranes and diffuse between cell compartments, which facilitates its signaling function (Zhang et al., 2019).

Several articles have stated that H_2_O_2_ can act as a key regulator in modulating the defense response of plants to various environmental stresses, such as salt stress (Silva et al., 2019), drought (Hossain and Fujita, 2013), high temperatures (Wu et al., 2015) and heavy metals (Wen et al., 2013). However, few studies show how they establish the criteria for selecting concentrations for exogenous application and what are the main mechanisms responsible for the increase in plant tolerance induced by H_2_O_2_ priming.

Sunflower (*Helianthus annuus* L.) is grown throughout the world. Since sunflower adapts to different edaphoclimatic conditions, it can be planted from Southern to Northern Brazil (Castro and Campos Leite, 2018). However, in regions affected by salinity, as Northeast Brazil, its growth and yield can be strongly reduced.

Thus, this study aimed to test different leaf spraying strategies with H_2_O_2_ for acclimation of the sunflower plants to salt stress, identifying the physiological and biochemical changes involved in this process.

## 2 Materials and methods

### 2.1 Experimental conditions

For this study, two experiments were carried out in the greenhouse of the Federal University of Recôncavo da Bahia, Cruz das Almas, BA, Brazil, using sunflower seeds of cultivar Agrobel 975 (AG 975).

### 2.2 First experiment (selection of treatments)

The seeds were placed to germinate in polyethylene trays containing washed sand and irrigated with half-strength Furlani's nutrient solution (Furlani, 1997). After the full expansion of the first pair of leaves, the seedlings were transferred to polyethylene pots and cultivated in hydroponic system containing 15 L of full-strength Furlani's nutrient solution where the treatments were distributed.

The experimental design used was completely randomized, with four repetitions. The treatments consisted of four concentrations of H_2_O_2_ (0.1; 1; 10; 100 mM) associated with different applications (1AP - one application 48 h before exposure to NaCl); 2AP - two applications (1AP + one application 7 days after exposure to NaCl) and 3AP - three applications (2AP + one application 14 days after exposure to NaCl). Two reference treatments were also added: control (without NaCl and leaf spraying with deionized water) and salt control (with 100 mM NaCl and leaf spraying with deionized water), totaling 14 treatments.

The leaf sprayings were performed in the early evening (at 6:00 p.m.), using a manual sprayer with a pre-compression pump and a 1.5 L reservoir. In the spraying solutions were added 0.025% Tween 20 (surfactant), to break the surface tension of the water and favor the penetration of H_2_O_2_ in the leaves (Gondim et al., 2012). Two days after the first leaf spraying, all nutrient solutions were replaced and the NaCl (100 mM) was added to establish salt stress.

At 35 days after sowing (DAS) the plants were harvested and partitioned into leaves, stems and roots, dried in an oven at 65 °C for 72 h, and then weighed in an analytical balance to quantify the dry masses of leaves (LDM), stem (SDM) and roots (RDM). With these data the total dry mass of the plants (TDM) was determined.

### 2.3 Second experiment

This experiment was carried out in randomized blocks with four repetitions, in structure similar to first experiment.

For this experiment, the treatment with H_2_O_2_ application that presented the highest dry mass yields (in leaves, stem and roots) in the previous experiment was selected. The three treatments tested were: control (without NaCl + leaf spraying with deionized water), salt control (with 100 mM NaCl + leaf spraying with deionized water) and 1 mM H_2_O_2_ (1AP) + 100 mM NaCl. To evaluate the behavior of the plants over time, two harvests were carried out: the first at 21 DAS and the second at 35 DAS.

In each harvest, fresh samples of the youngest pair of fully expanded leaves were collected for pigment, variables related to leaf water status and electrolyte leakage determinations (10 discs of 0.8 cm diameter for each assessment). Samples of the same leaves and the younger third of the root system also were collected, frozen, lyophilized and ground to a powder for organic solutes, antioxidant enzyme activity and lipid peroxidation analyses. The rest of the plant material was dried in an oven at 65 °C for 72 h to determine the shoot dry mass (ShDM). The dried material of leaves and roots was ground for inorganic solutes determination.

### 2.4 Measurements of the gas exchange and photosynthetic pigments content

Gas exchange evaluations were performed, at 21 and 35 DAS, on the youngest pair of fully expanded leaves, using an infrared gas analyzer - IRGA, model Li-6400XT (Li-Cor, Lincoln, NE, USA). The net CO_2_ assimilation rate (P_N_), transpiration (E) and stomatal conductance (gs) were determined. The levels of photosynthetic pigments chlorophyll *a* (Chl *a*), chlorophyll *b* (Chl *b*), chlorophylls *a* + *b* (Chl *a* + *b*) and carotenoids (Car) were extracted in ethanol (95%) and quantified by spectrophotometry at 664, 649 and 470 nm, using the equations proposed by Lichtenthaler and Buschmann (2001).

### 2.5 Water status and electrolyte leakage

In the same pair of leaves used for photosynthetic evaluations were analyzed: the relative water content (RWC), water saturation deficit (WSD), water content at saturation (WCS), leaf succulence (SUC), sclerophylly index (SI) and electrolyte leakage (EL). For this, ten leaf discs (diameter 0.8 cm) were sampled and immediately weighed to obtain the fresh mass (FM). Then, they were immersed in distilled water in Petri dishes for 12 h at 25 °C under dark, blotted on filter paper, and the turgid mass (TM) was determined. The discs were dried in an oven at 75 °C for 48 h and the dry mass (DM) was obtained. The RWC, WSD, WCS, SUC and SI were calculated using the following equations: RWC (%) = [(FM - DM)/(TM - DM) × 100]; WSD (%) = [(TM - FM)/(TM - DM) × 100]; WCS (mg H_2_O mg^−1^ DM) = [(TM - FM)/DM]; SUC (mg H_2_O cm^−2^) = [(FM - DM)/LA] and SI (mg cm^−2^) = (DM/LA), where LA is the leaf area of the 10 leaf discs (diameter 0.8 cm) (Bacelar et al., 2004).

Electrolyte leakage was calculated using ten leaf discs (diameter 0.8 cm) placed in test tubes containing 10 mL deionized water. Tubes were incubated in a shaking water bath at 25 °C for 24 h and the initial electrolyte leakage (EL_initial_) of the medium was measured by electrical conductivity. After, samples were boiled at 100 °C for 1 h to release all electrolytes and cooled to 25 °C for measurement of the final electrolyte leakage (EL_final_). The eletrolyte leakage (percentage of membrane damage, MD) was calculated according Silva et al. (2015), using the formula: EL (%) = [(EL_initial_/EL_final_) × 100].

### 2.6 Inorganic solutes content

For determination of the contents of sodium (Na^+^), potassium (K^+^) and chloride (Cl^−^), the extracts of oven-dried samples of leaves and roots were prepared in deionized water following the methodology adapted by Cova et al. (2016).

The contents of Na^+^ and K^+^ were determined by flame photometry model Q498M2 (Quimis, Diadema, SP, BR), and Cl^−^ content was determined in a spectrophotometer UV-VIS model 2000 UV (Bel Engineering, Piracicaba, SP, BR), as described by Cova et al. (2016).

### 2.7 Organic solutes content

For determination of the contents of soluble carbohydrates, free amino acids, free proline and soluble proteins, the extracts were prepared in buffer solution (100 mM potassium phosphate, pH 7.0, 0.1 mM EDTA), using the powder of lyophilized leaves and roots samples (Azevedo Neto et al., 2009).

The determination of the content of soluble carbohydrates was carried out at 490 nm, using the phenol-sulfuric acid method (Dubois et al., 1956). Free amino acids were determined at 570 nm, using the ninhydrin method (Yemm and Cocking, 1955). To determine the free proline content, the 520 nm acid ninhydrin method was used (Bates et al., 1973). Soluble proteins were determined at 595 nm by the protein-dye binding method (Bradford, 1976), using bovine albumin as a standard.

### 2.8 Antioxidant enzyme activity and lipid peroxidation

The extracts for determining the activity of antioxidant enzymes and lipid peroxidation were prepared in a similar way to organic solutes (Azevedo Neto et al., 2009).

The activity of superoxide dismutase (SOD) (EC 1.15.1.1) was determined by measuring its ability to inhibit the photochemical reduction of nitro blue tetrazolium chloride - NBT (Giannopolitis and Ries, 1977) and the results expressed in U g^−1^ of dry mass (DM). One unit of SOD activity (U) was defined as the amount of enzyme needed to cause 50% inhibition of the NBT photoreduction rate. The activity of catalase (CAT) (EC 1.11.1.6) was measured from the decrease in the concentration of H_2_O_2_ following the method of Beers and Sizer (1952), modified by (Azevedo Neto et al., 2005) and expressed in μmol H_2_O_2_ min^−1^ g^−1^ DM. The evaluation of the activity of ascorbate peroxidase (APX) was measured from the oxidation of ascorbate, following the method described by Nakano and Asada (1981). For this enzyme, the results were expressed in μmol H_2_O_2_ min^−1^ g^−1^ DM, considering that 2 mol of ascorbate are needed to reduce 1 mol of H_2_O_2_ (McKersie and Leshem, 1994).

Lipid peroxidation (LP) was determined by measuring the content of malondialdehyde (MDA) produced by the reaction of thiobarbituric acid (Heath and Packer, 1968), and the results were expressed as μmol MDA g^−1^ DM.

### 2.9 Statistical analysis

All data from both experiments were submitted to analysis of variance (ANOVA). In the first experiment, the means of the variables were compared by Scott-Knott's test (p ≤ 0.05), and in the second experiment, the means were compared by Tukey's test (p ≤ 0.05), using the Sisvar statistical program (Ferreira, 2011).

## 3 Results

### 3.1 First experiment

The results showed that there was a significant difference between the treatments applied (p ≤ 0.01) for the growth variables analyzed (Table 1). Salinity reduced plant growth, but H_2_O_2_ priming via leaf spraying (in some treatments) was able to reduce the deleterious effects of salinity and improve plant tolerance to salt stress.

**Table 1:** Summary of result of the analysis of variance and the Scott-Knott test for the parameters analyzed in the first experiment on sunflower plants, at 35 days of cultivation.

Sources of variations	LDM	SDM	RDM	TDM
Treatments	∗∗	∗∗	∗∗	∗∗
CV (%)	10.83	10.82	11.78	5.93
Treatments	Leaves	Stem	Roots	Total
	Dry mass (% of control)			
T1 - control	100	100	100	100
T2 - salt control	40.5 b	25.5 b	45.1 b	34.8 c
T3 - 0.1 mM H_2_O_2_ (AP1)	35.9 b	28.6 b	50.6 b	36.1 c
T4 - 1 mM H_2_O_2_ (AP1)	47.8 a	34.7 a	59.9 a	44.8 a
T5 - 10 mM H_2_O_2_ (AP1)	40.0 b	23.8 b	45.1 b	33.9 c
T6 - 100 mM H_2_O_2_ (AP1)	45.4 a	33.0 a	42.1 b	39.1 b
T7 - 0.1 mM H_2_O_2_ (AP2)	38.3 b	27.3 b	45.4 b	35.0 c
T8 - 1 mM H_2_O_2_ (AP2)	48.4 a	31.9 a	49.1 b	41.1 a
T9 - 10 mM H_2_O_2_ (AP2)	44.2 a	33.0 a	50.3 b	41.0 a
T10 - 100 mM H_2_O_2_ (AP2)	43.8 a	28.1 b	50.9 b	38.4 b
T11 - 0.1 mM H_2_O_2_ (AP3)	36.2 b	26.2 b	46.7 b	34.2 c
T12 - 1 mM H_2_O_2_ (AP3)	42.9 a	32.0 a	46.7 b	38.9 b
T13 - 10 mM H_2_O_2_ (AP3)	36.7 b	35.3 a	47.8 b	38.7 b
T14 - 100 mM H_2_O_2_ (AP3)	37.4 b	27.6 b	46.3 b	35.1 c

∗∗ Significant (p ≤ 0.01). Means followed by the same letter, in the column, do not differ statistically from each other by the Scott-Knott's test (p ≤ 0.05). LDM (leaves dry mass), SDM (stem dry mass), RDM (roots dry mass) and TDM (total dry mass).

The salt control treatment (T2) showed decreases in LDM, SDM, RDM and TDM of 59.5, 74.5, 54.9 and 65.2%, respectively, when compared to plants of the control treatment (T1) (Table 1). In contrast, some H_2_O_2_-primed treatments showed on average an increase of 12% in LDM (T4, T6, T8, T9, T10 and T12), 31% in SDM (T4, T6, T8, T9, T12 and T13), 33% in the RDM (T4), and 23% in the TDM (T4, T8 and T9) when compared with the plants of the T2 treatment.

The selection of treatments was based on biomass production, considering all plant partitions (LDM, SDM, RDM and TDM), therefore T4 treatment (1.0 mM H_2_O_2_ 1AP) was selected for its greater effectiveness in mitigating the negative effects of the salinity and, consequently increasing the tolerance of sunflower plants (Table 1).

### 3.2 Second experiment

The result of the analysis of variance showed significant differences among the tested treatments for the dry mass yield, content of photosynthetic pigments, variables for evaluation of water status, leakage of electrolytes, content of inorganic and organic solutes and enzyme activity in both leaves and roots (at 21 and 35 days) (Supplementary Table 1). For CO_2_ assimilation, the significant difference occurred only at 35 days, while stomatal conductance and transpiration were not significantly affected by the treatments applied (Supplementary Table 1).

**Figure 1:**
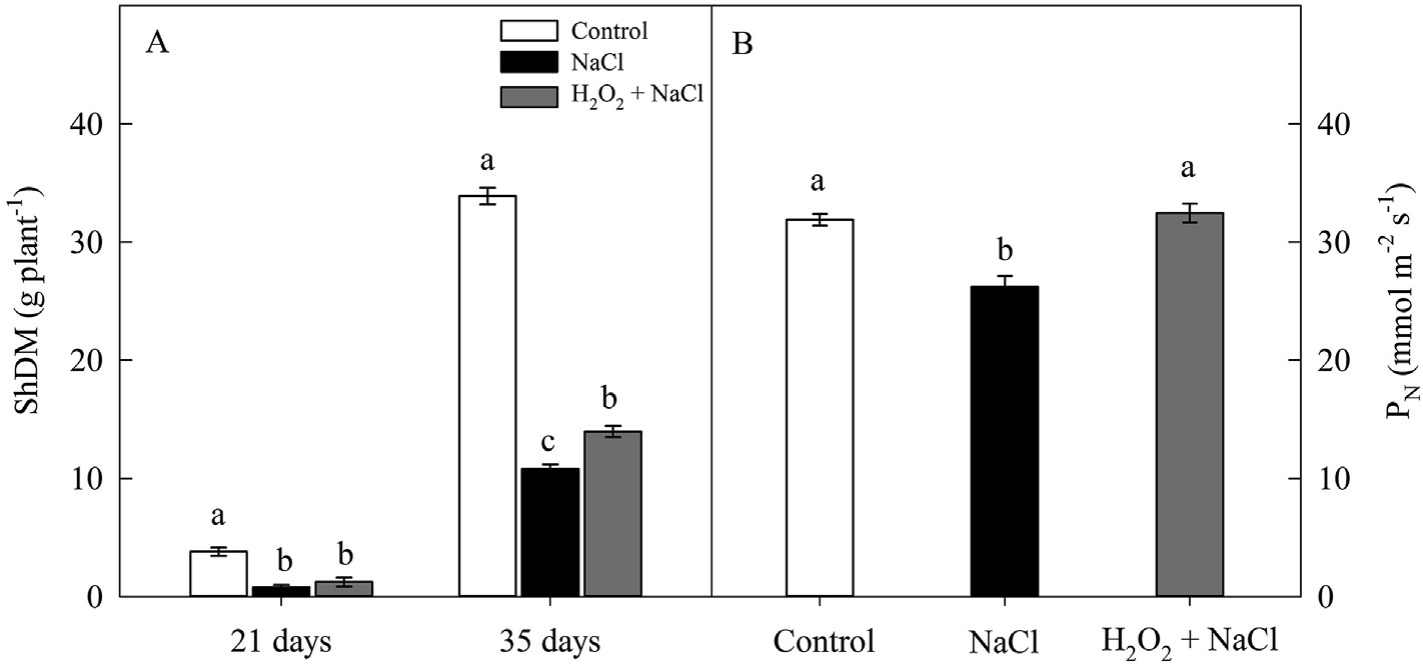
Effect of salt stress (100 mM NaCl) and leaf spraying with H_2_O_2_ (1 mM H_2_O_2_ 1AP^∗^) on the shoot dry mass (ShDM) (at 21 and 35 days) (A) and on the net CO_2_ assimilation rate (P_N_) (at 35 days) (B) of sunflower plants grown in nutrient solution. Means of four repetitions ±standard error. Means followed by the same letters, on each date, do not differ statistically from each other, using the Tukey's test (p ≤ 0.05). ^∗^1AP - one application by leaf spraying 48 h before exposure to NaCl.

Salt stress reduced the ShDM of sunflower plants by 73% (at 21 days). However, at 35 days, this reduction was less pronounced in H_2_O_2_-primed plants (Figure 1A). In this harvest, salt stress reduced ShDM by 68% in non-primed plants when compared to control. On the other hand, ShDM in H_2_O_2_-primed plants was 29% higher than in non-primed ones (Figure 1A). At 35 days, the P_N_ was reduced by 18% in plants under NaCl treatment, in contrast to H_2_O_2_-primed plants, where the P_N_ was similar to control treatment (Figure 1B).

Associated with this result, at 35 days, salinity also reduced P_N_ by 18% in plants non-primed with H_2_O_2_. In contrast, even under salt stress, H_2_O_2_ priming was able to maintain the P_N_ of sunflower plants at levels similar to that of control treatment (Figure 1B).

**Figure 2:**
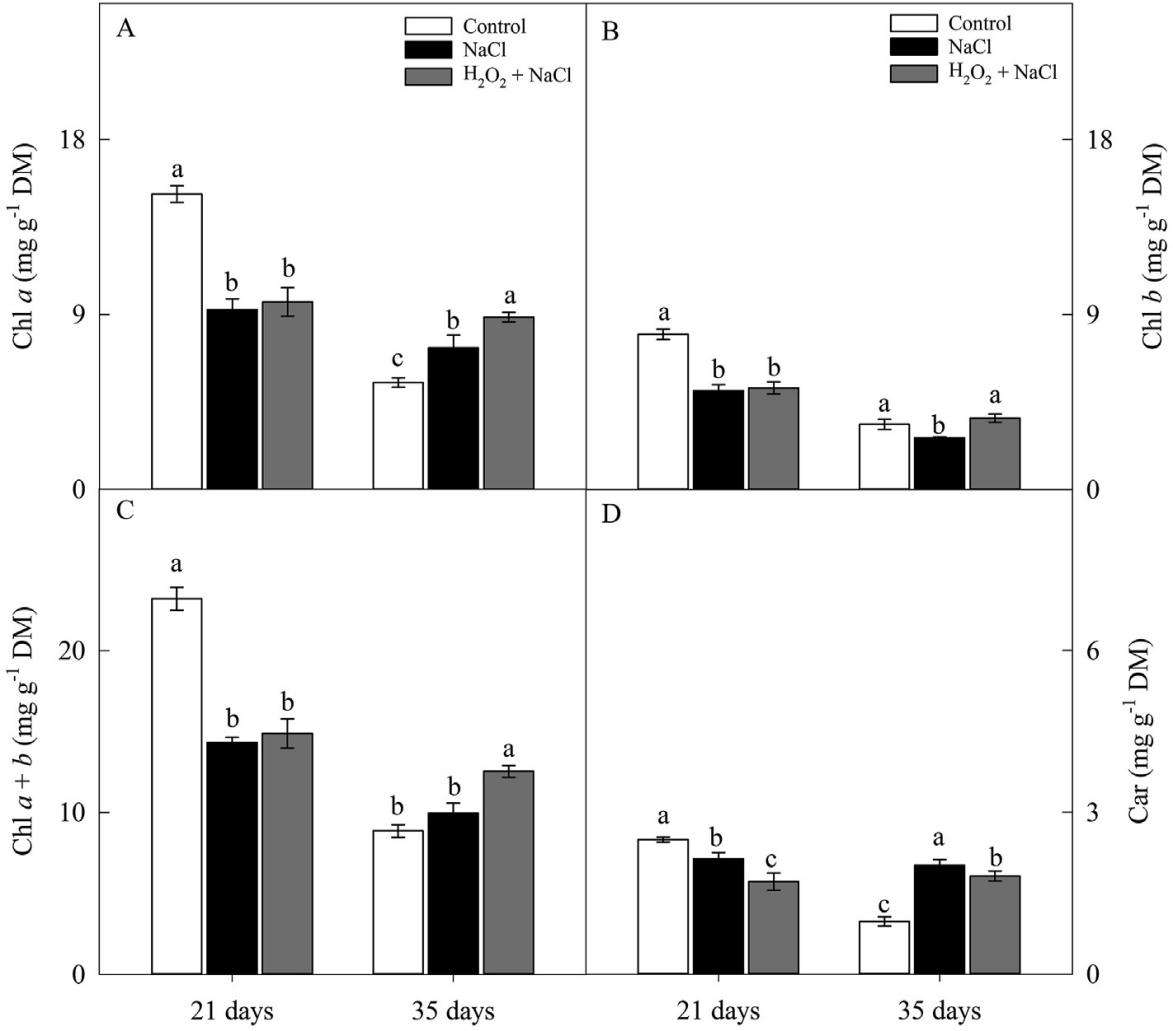
Effect of salt stress (100 mM NaCl) and leaf spraying with H_2_O_2_ (1 mM H_2_O_2_ 1AP^∗^) on the content of chlorophyll a (Chl *a*) (A), chlorophyll b (Chl *b*) (B), chlorophyll *a* + *b* (Chl *a* + *b*) (C) and carotenoids (Car) (D) of sunflower plants grown in nutrient solution, at 21 and 35 days. Means of four repetitions ±standard error. Means followed by the same letters, on each date, do not differ statistically from each other, using the Tukey's test (p ≤ 0.05). ^∗^1AP - one application by leaf spraying 48 h before exposure to NaCl.

At 21 days, the levels of photosynthetic pigments (Chl *a*, Chl *b*, Chl *a* + *b* and Car) were, on average, 39, 39, 45 and 33% lower in plants under salt stress when compared to the plants in the control treatment, respectively (Figure 2). However, at 35 days, the contents of Chl *a* and Chl *a* + *b* were, respectively, 39% and 33% higher in H_2_O_2_-primed plants in comparison to all non-primed treatments (Figure 2A and C). Under salt stress, H_2_O_2_ priming also increased the Chl *b* contents by 38% compared to plants of the salt control treatment (Figure 2B). In opposite to the results verified at 21 days, Car content (at 35 days) was 11% higher in the salt control treatment (Figure 2D).

**Figure 3:**
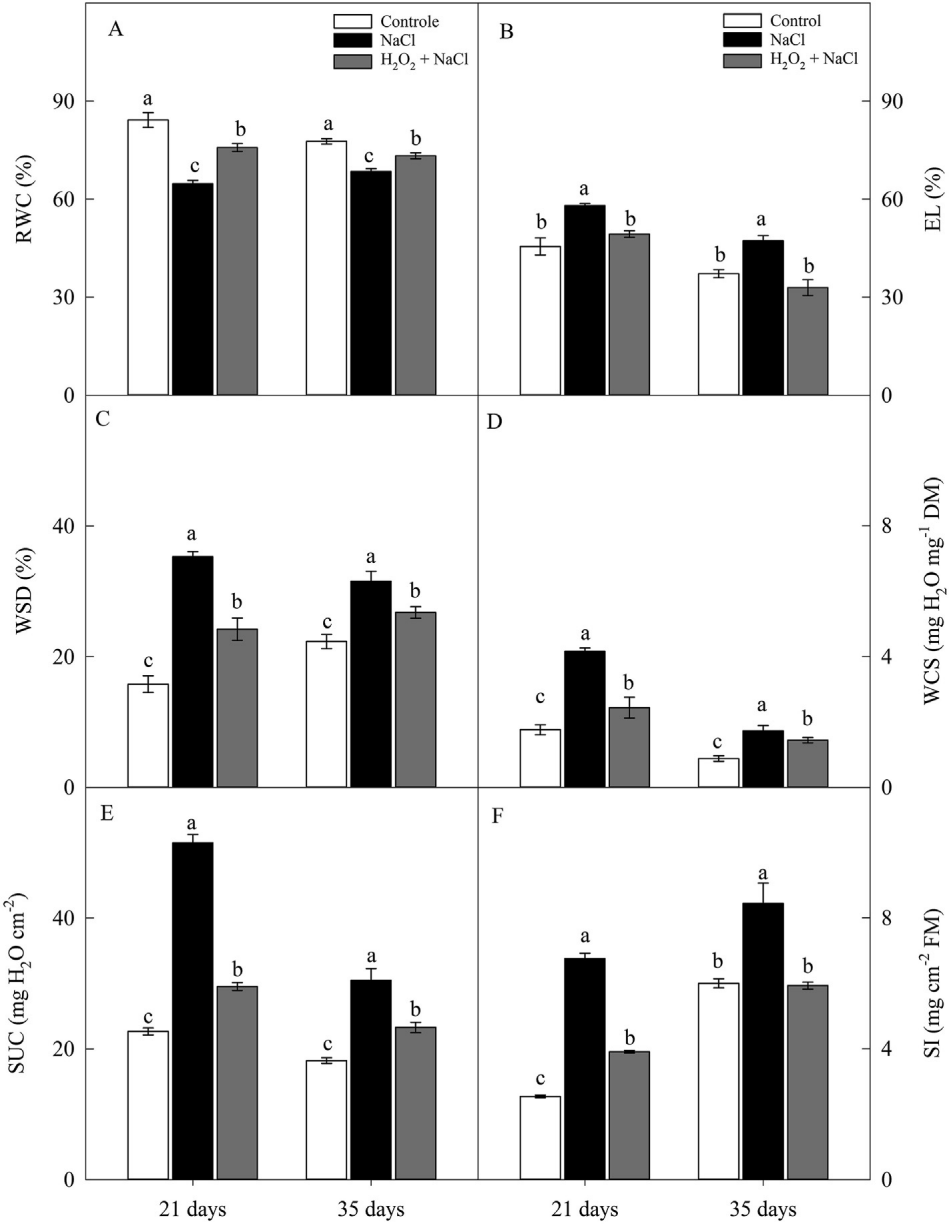
Effect of salt stress (100 mM NaCl) and leaf spraying with H_2_O_2_ (1 mM H_2_O_2_ 1AP^∗^) on the relative water content (RWC) (A), electrolyte leakage (EL) (B), water saturation deficit (WSD) (C), water content at saturation (WCS) (D), leaf succulence (SUC) (E) and sclerophylly index (SI) (F) of the leaves of sunflower plants grown in nutrient solution, at 21 and 35 days. Means of four repetitions ±standard error. Means followed by the same letters, on each date, do not differ statistically from each other, using the Tukey's test (p ≤ 0.05). ^∗^1AP - one application by leaf spraying 48 h before exposure to NaCl.

Salt stress significantly reduced the RWC of sunflower leaves, however, as with the ShDM results, this decrease was less pronounced in H_2_O_2_-primed plants (Figure 3A). Therefore, the RWC of primed plants was 17% (21 days) and 7% (35 days) higher than in non-primed ones (Figure 3A).

The EL of salt control plants was about 22% (21 days) and 35% (35 days) more than in comparison to other treatments (Figure 3B). In contrast, the EL of H_2_O_2_-primed plants, even under salt stress, remained at levels similar to those of the plants of the control treatment (Figure 3B). Salt stress increased the EL by 22% and 35% at 21 and 35 days, respectively, only in plants non-primed with H_2_O_2_ (Figure 3B).

The salt stress increased the WSD, WCS, SUC and SI of the plants, except the SI in the treatment H_2_O_2_-primed (at 35 days), however this increase was much more expressive in non-primed plants (Figure 3C, D, E and F). At 21 and 35 days, salinity in non-primed plants increased WSD (by 123% and 41%), WCS (by 136% and 96%), SUC (by 127% and 67%) and SI (by 166% and 41%), respectively when compared to control plants. For the same periods (21 and 35 days), the increases in H_2_O_2_-primed plants were, respectively: WSD (53% and 20%), WCS (39% and 63%), SUC (30% and 28%), and SI(53%, only at 21 days) (Figure 3C, D, E and F).

**Figure 4:**
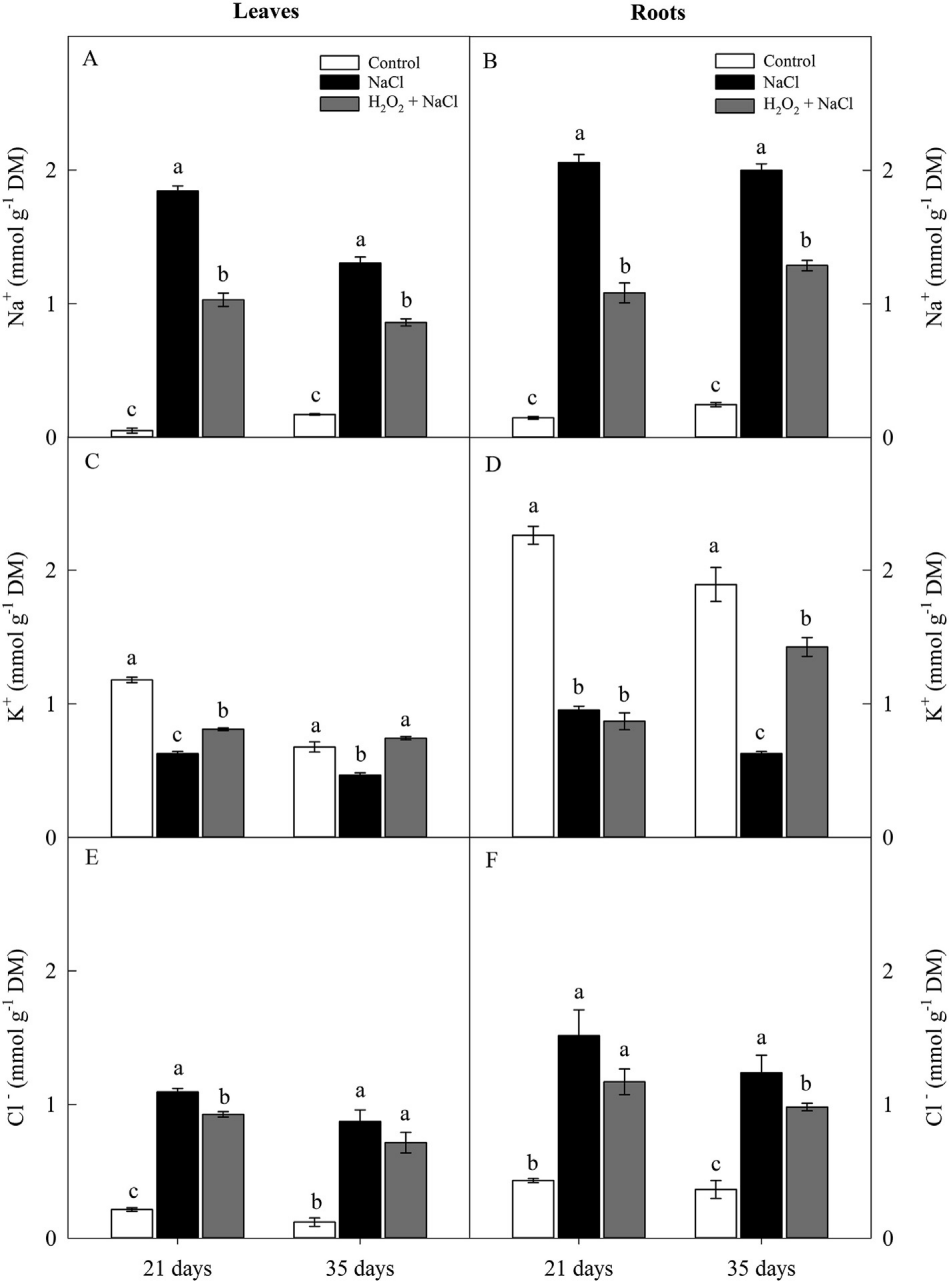
Effect of salt stress (100 mM NaCl) and leaf spraying with H_2_O_2_ (1 mM H_2_O_2_ 1AP^∗^) on the contents of Na^+^ (A and B), K^+^ (C and D), Cl^−^ (E and F) in leaves and roots, respectively, of sunflower plants grown in nutrient solution, at 21 and 35 days. Means of four repetitions ±standard error. Means followed by the same letters, on each date, do not differ statistically from each other, using the Tukey's test (p ≤ 0.05). ^∗^1AP - one application by leaf spraying 48 h before exposure to NaCl.

The contents of Na^+^ and Cl^−^ in sunflower leaves and roots also increased under conditions of salt stress in both periods of evaluation. However, H_2_O_2_ priming significantly reduced the levels of Na^+^ in the leaves and roots (at 21 and 35 days) and the levels of Cl^−^ in the leaves (at 21 days) and roots (at 35 days) (Figure 4A, B, E and F). Analyzing both periods together (21 and 35 days), the salt stress in non-primed plants increased, on average 14.1-fold and 10.3-fold (Na^+^) and 5.9-fold and 3.4-fold (Cl^−^) in the leaves and roots, respectively, compared to control plants. In contrast, H_2_O_2_ priming reduced the Na^+^ content on average 40% (in leaves) and 42% (in roots) compared to non-primed plants (Figure 4A, B, E and F).

The K^+^ contents were strongly reduced by the salt stress in the leaves (47% and 31%) and roots (58% and 67%) in the non-primed plants when compared with the plants of the control ones, at 21 and 35 days, respectively (Figure 4C and D). In primed plants, except for roots (at 21 days), the H_2_O_2_ increased K^+^ content in the leaves by 29% and 59% (21 and 35 days), respectively, and in the roots this increase was of 128 %, at 35 days (Figure 4C and D).

**Figure 5:**
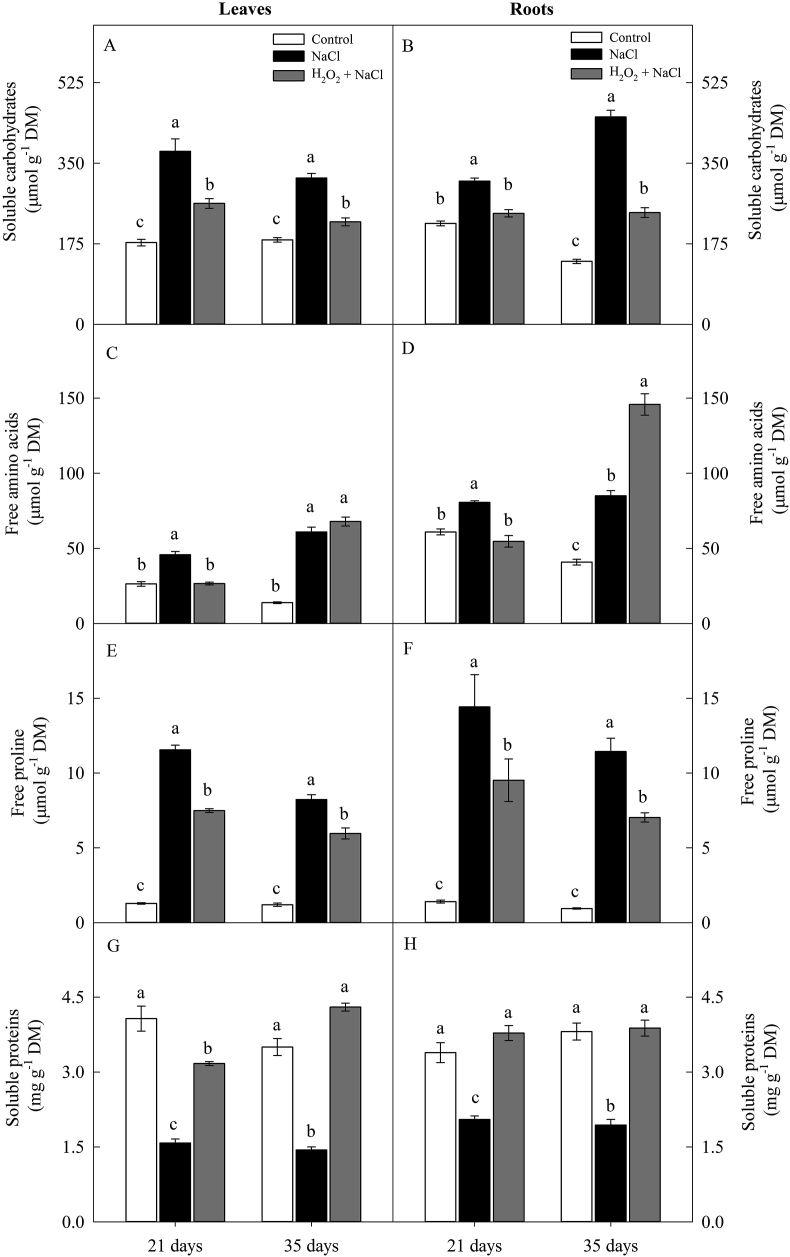
Effect of salt stress (100 mM NaCl) and leaf spraying with H_2_O_2_ (1 mM H_2_O_2_ 1AP^∗^) on the levels of soluble carbohydrates (A and B), free amino acids (C and D), free proline (E and F), and soluble proteins (G and H) in leaves and roots, respectively, of sunflower plants grown in nutrient solution, at 21 and 35 days. Means of four repetitions ±standard error. Means followed by the same letters, on each date, do not differ statistically from each other, using the Tukey's test (p ≤ 0.05). ^∗^1AP - one application by leaf spraying 48 h before exposure to NaCl.

In both periods (21 and 35 days), salinity increased, on average, the contents of soluble carbohydrates, free amino acids and free proline in leaves (1.9-fold, 2.6-fold and 7.9-fold) and roots (2.1-fold, 1.6-fold and 11-fold) of the non-primed plants, compared to the control treatment (Figure 5A, B, C, D, E and F). On the other hand, in the plants primed with H_2_O_2_ the average increase in the levels of soluble carbohydrates and free proline, in both periods, were 1.3-fold and 5.4-fold (leaves) and 1.3-fold and 7-fold (roots), respectively, compared to the control treatment (Figure 5A, B, C, D, E and F).

At 21 days, H_2_O_2_ priming maintained the levels of free amino acids, in leaves and roots, similar to those found in the plants of the control treatment (Figure 5C and D). In contrast, at 35 days, H_2_O_2_-primed plants showed an increase in the content of free amino acids of 4.8-fold (leaves), compared to the control treatment, and of 2.7-fold and 1.7-fold (roots) in comparison to non-primed treatments under control conditions and under salt stress conditions, respectively (Figure 5C and D).

Salt stress reduced the soluble protein content of leaves and roots by 61% and 40% (21 days) and 59% and 49% (35 days), respectively, in plants non-primed with H_2_O_2_ in comparison to the control treatment (Figure 5G and H). In contrast to these results, the soluble protein content, in primed plants, was similar to the control treatment, except in the leaves (at 21 days) (Figure 5G and H).

**Figure 6:**
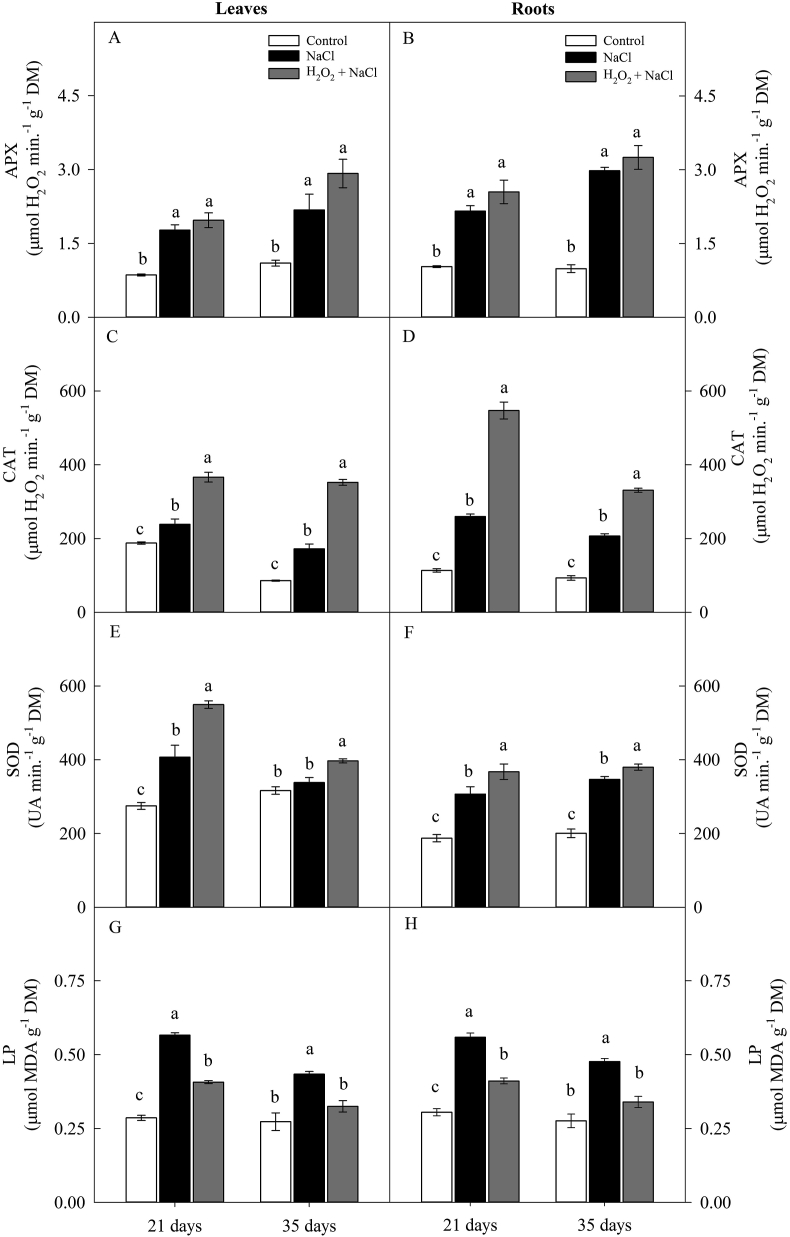
Effect of salt stress (100 mM NaCl) and leaf spraying with H_2_O_2_ (1 mM H_2_O_2_ 1AP^∗^) on the activity of ascorbate peroxidase (APX) (A and B), catalase (CAT) (C and D), superoxide dismutase (SOD) (E and F), and lipid peroxidation (LP) (G and H) in leaves and roots, respectively, of sunflower plants grown in nutrient solution, at 21 and 35 days. Means of four repetitions ±standard error. Means followed by the same letters, on each date, do not differ statistically from each other, using the Tukey's test (p ≤ 0.05). ^∗^1AP - one application by leaf spraying 48 h before exposure to NaCl.

In general, salt stress increased the activity of antioxidant enzymes (APX, CAT and SOD), except, SOD activity on leaves (at 35 days) of the non-primed plants (Figure 6A, B, C, D, E and F). Regardless of H_2_O_2_ priming, salt stress increased the APX activity of leaves and roots on an average 2.2-fold and 2.3-fold (21 days) and 2.3-fold and 3.1-fold (35 days), respectively, when compared to control treatment (Figure 6A and B).

In general, the increase of the CAT and SOD activities were more expressive in H_2_O_2_-primed plants. Under salinity, CAT and SOD activities on leaves were increased by 53% and 35% (at 21 days) and 48% and 17% (at 35 days), while in the roots these increases were 110% and 19% (21 days) and 60% and 9% (35 days) when compared to non-primed treatment (Figure 6C, D, F and F).

In the same period, the increase of the CAT activity in the plants H_2_O_2_ primed plants was 95% and 309% (leaves) and 381 and 255% (roots), respectively, when compared to the control treatment (Figure 6C and D).

At 21 days, the LP in the leaves and roots were significantly increased by salt stress, but these increases were less pronounced in H_2_O_2_-primed plants (Figure 6G and H). The LP in the leaves and roots of the plants under salt stress and non-primed was about 98 and 95% higher than in the plants of the control treatment. However, in the same conditions, H_2_O_2_ priming reduced the LP in the leaves (28%) and roots (31%), when compared to non-primed plants (Figure 6G and H).

At 35 days, the salt stress increased the LP of the leaves and roots only in the non-primed plants (59 and 73%, respectively) in comparison to the plants of the control treatment. In this period, H_2_O_2_ priming reduced the LP maintaining levels similar to those of the control plants (Figure 6G and H).

## 4 Discussion

As expected, the results of the first experiment showed that salt stress reduced the growth of sunflower plants. However, some treatments, especially, leaf spraying with 1 mM H_2_O_2_ applied 48 h before exposure to salt stress was able to increase dry mass production in all parts of sunflower plants, improving its tolerance to salt stress. In this experiment, the results also showed that the H_2_O_2_ application strategy via leaf spraying (concentration and number of applications) was different from those found in other studies. These results indicate that it is necessary to carry out preliminary tests to identify the best application strategy for each crop. Gondim et al. (2012) stated that from tests performed (data not shown) the best concentration used for leaf spraying was 10 mM H_2_O_2_ to maize plants applied only 48 h before exposure to salt. While Semida (2016), using another application strategy, stated that the 1 mM of H_2_O_2_ (applied at 20, 40 and 60 days after transplanting) was the one that improved the responses of onion plants to salt stress.

In the second experiment, the analysis of the results showed that the reduction of growth and the net CO_2_ assimilation rate in sunflower plants cultivated under salt stress did not occur due to stomatal limitation. However, the increase in the concentration of toxic ions (Na^+^ and Cl^−^) may have been the main factor for the reduction of growth and photosynthesis. Studies affirm that the reduction of the RuBisCo carboxylation efficiency can be directly related to the accumulation of Na^+^ and Cl^−^ in photosynthetic tissues (Melo et al., 2020).

The increase in ShDM and P_N_ in primed plants (Figure 1A) can be directly associated with the signaling role of H_2_O_2_. The H_2_O_2_-induced cross-tolerance mechanism is based on the triggering of several highly complex reactions that are mainly related to the pathways of mitogen-activated protein kinases (MAPKs route) and the route of calcium-dependent protein kinases (CDPKs) (Hossain et al., 2015; Kurusu et al., 2015).

Several studies have shown that H_2_O_2_ is capable of increasing the tolerance of maize plants (Azevedo Neto et al., 2005; Gondim et al., 2012), onion (Semida, 2016), pistachio (Bagheri et al., 2019) and basil (Silva et al., 2019).

The reduction in pigment content by salt stress observed at 21 days (Figure 2) can be attributed to the increase in chlorophyllase activity, which is the main enzyme responsible for the degradation of chlorophylls (Taïbi et al., 2016). In contrast, the increase observed at 35 days, in H_2_O_2_-primed plants may be because the chloroplast protein associated with chlorophyll is unexcited, facilitating the process of chlorophyll extraction under salt stress conditions (Liang et al., 2018). Cova et al. (2019) and Silva et al. (2019) also found that salinity increased the Chl *a* content in noni (*Morinda citrifolia*) and basil (*Ocimum basilicum*) plants, respectively.

The increase observed at 35 days in the carotenoid content in plants under salt stress (Figure 2D) occurred as a plant protection mechanism, dissipating the excess energy accumulated by stress in the form of heat through the xanthophyll cycle. Salt stress disrupts the balance between photosynthetic electron transport and Calvin-Benson's cycle reactions, leading to over-reduction and excess energy within of the thylakoids (Cerqueira et al., 2019). Carotenoids are integral constituents of thylakoid membranes acting as accessory pigments in the capture of light and as photoprotective agents in the dissipation of excess absorbed light (Maoka, 2020). In primed plants, on the other hand, the reduction of the deleterious effects of salt stress associated with the increase in P_N_ may have contributed to the reduction of excess energy in thylakoids and maintaining the balance of carotenoid production.

Our results also show that, for the non-primed treatment with H_2_O_2_, the salt stress reduced the RWC and increased the WSD, WCS, SUC and SI in the leaves (Figure 3C, D, E and F). In contrast, H_2_O_2_ priming improved the water status of the plants, reducing the water loss, as indicated by increase of WSD and WCS. High values of SI indicate an increase in the leaf thickness. Some authors claim that an increase in SI is a mechanism developed to increase the resistance to the diffusion of water in the leaf and, consequently, to minimize the water losses (Cunningham and Strain, 1969). As a consequence of this mechanism, there is also an increase in SUC, which is a variable that indicates the amount of water per unit leaf area. Some authors claim that the increase in SUC, besides maintaining water storage, can be an important mechanism for diluting toxic ions (Cova et al., 2016; Silva et al., 2019).

The increase in the levels of Na^+^ and Cl^−^ and reduction of K^+^ in the cytosol, verified in plants under salt stress can cause several physiological disturbances and cause an ion imbalance (Figure 4). However, in H_2_O_2_-primed plants, the decrease in Na^+^ and Cl^−^ levels and the increase in K^+^ content in both leaves and roots suggests that H_2_O_2_ was able to trigger physiological mechanisms of Na^+^ and Cl^−^ exclusion and reduction of K^+^ efflux from tissues, improving the ion homeostasis and increasing the salt tolerance in sunflower plants. Some studies have stated that H_2_O_2_ can induce an increase in the K^+^/Na^+^ ratio and reduce the Cl^−^ content in plants under salt stress (Silva et al., 2019).

H_2_O_2_ can activate Ca^2+^ input channels to the cytosol and this, in turn, is one of the main mechanism responsible for the activation of the SOS (salt overly sensitive) pathway, formed by the SOS1, SOS2 and SOS3 proteins and responsible for the extrusion of Na^+^ from cytosol (Niu and Liao, 2016).

The expressive increase in the contents of soluble carbohydrates, free amino acids and free proline salt-induced in the leaves and roots of non-primed plants can be considered a mechanism to protect plants from salt stress (Reddy et al., 2017). Under salt stress, plants accumulate organic compounds of low molecular mass, whose main functions are to help maintain water status, protect the cells against oxidative damage, and act as signaling agents during stress (Azevedo Neto et al., 2009).

The increase in the content of soluble proteins in primed plants may be related to the signaling role of H_2_O_2_ in the expression of specialized proteins that respond to salt, including antioxidant enzymes (Hossain et al., 2015; Niu and Liao, 2016; Černý et al., 2018).

Under salt stress, the excess of free energy verified in the plants non-primed with H_2_O_2_, associated with the increase in the concentration of toxic ions induced by salt stress can have contributed to the increase in the ROS production and, consequently, increasing the damage to the plasma membrane, indicated by the increase in EL and of LP. In contrast, plants H_2_O_2_-primed were similar to the of the control plants, indicating once again a reduction in the NaCl-induced negative effects (Figures 3B, 6G and H).

This increase in the production of ROS (under stress conditions) can lead to an imbalance of redox homeostasis, characterizing oxidative stress, and consequently causing disturbances in cell structure and metabolism (Khan et al., 2019). However, H_2_O_2_ priming induces an increase in antioxidant activity by increasing the level of transcripts and expression of antioxidant enzyme genes, such as superoxide dismutase, catalase, ascorbate peroxidase, guaiacol peroxidase and others (Azevedo Neto et al., 2005; Gondim et al., 2012; Hossain et al., 2015; Savvides et al., 2016). The increased expression of these enzymes can significantly contribute to the maintenance of redox homeostasis, acting as one of the key mechanisms to mitigate the deleterious effect of salt stress (Azevedo Neto et al., 2005).

Among the antioxidant enzymes, catalase stands out for the significant increase in its activity in both leaves and roots for the both evaluation periods. The results observed by in the present study confirm the hypothesis suggested by Azevedo Neto et al. (2005) and Gondim et al. (2012) that the increase of catalase activity induced by H_2_O_2_ priming play a key role in ROS detoxification, increasing the tolerance of plants to salt stress.

## 5 Conclusions

Our results show that the H_2_O_2_ priming via leaf spraying can increase the tolerance of plants to salt stress, mainly by the balance of ion homeostasis (by reducing the levels of Na^+^ and Cl^−^ and increasing the levels of K^+^) and homeostasis redox (due to increased antioxidant activity, mainly catalase).

## Declarations

### Author contribution statement

Petterson Costa Conceição Silva：Conceived and designed the experiments; Performed the experiments; Analyzed and interpreted the data; Contributed reagents, materials, analysis tools or data; Wrote the paper.

André Dias de Azevedo Neto：Conceived and designed the experiments; Performed the experiments; Analyzed and interpreted the data; Contributed reagents, materials, analysis tools or data; Wrote the paper.

Hans Raj Gheyi：conceived and designed the experiments; analyzed and interpreted the data; contributed reagents, materials, analysis tools or data; wrote the paper.

Rogério Ferreira Ribas：performed the experiments; contributed reagents, materials, analysis tools or data.

Caroline Rastely dos Reis Silva：performed the experiments; wrote the paper.

Alide Mitsue Watanabe Cova：analyzed and interpreted the data; wrote the paper.

### Funding statement

This work was supported by Coordenação de Aperfeiçoamento de Pessoal de Nível Superior (10.13039/501100002322CAPES), Conselho Nacional de Desenvolvimento Científico e Tecnológico (10.13039/501100003593CNPq), and the Fundação de Amparo à Pesquisa do Estado da Bahia (10.13039/501100006181FAPESB).

### Competing interest statement

The authors declare no conflict of interest.

### Additional information

Supplementary content related to this article has been published online at https://doi.org/10.1016/j.heliyon.2020.e05008.
