# Supplementary material for: Salt-tolerance induced by leaf spraying with H2O2 in sunflower is related to the ion homeostasis balance and reduction of oxidative damage
Source: Heliyon. 2020 Sep 21;6(9):e05008. doi: 10.1016/j.heliyon.2020.e05008 (PMC7509778; doi:10.1016/j.heliyon.2020.e05008)
Supplement: Supplementary Table [file mmc1.docx]

**Supplementary Table 1.** Summary of the Fisher’s test for the parameters analyzed in the second experiment using sunflower plants, at 21 and 35 days of cultivation.

| Parameters | 21 | 35 |
| --- | --- | --- |
|  | days | |
| ShDM (g plant^-1^) | ** | ** |
| P_N_ (µmol CO_2_ m^-2^ s^-1^) | ns | ** |
| E (mmol H_2_O m^-2^ s^-1^) | ns | ns |
| gs (mol H_2_O m^-2^ s^-1^) | ns | ns |
| Chl *a* (mg g^-1^ DM) | ** | ** |
| Chl *b* (mg g^-1^ DM) | ** | ** |
| Chl *a* + *b* (mg g^-1^ DM) | ** | ** |
| Car (mg g^-1^ DM) | ** | ** |
| RWC (%) | ** | ** |
| EL (%) | ** | ** |
| WSD (%) | ** | ** |
| WCS (mg H_2_O mg^-1^ DM) | ** | ** |
| SUC (mg H_2_O cm^-2^) | ** | ** |
| SI (mg DM cm^-2^) | ** | ** |
| Na^+^ _(leaves)_ (mmol g^-1^ DM) | ** | ** |
| K^+^ _(leaves)_ (mmol g^-1^ DM) | ** | ** |
| Cl^-^ _(leaves)_ (mmol g^-1^ DM) | ** | * |
| Na^+^ _(roots)_ (mmol g^-1^ DM) | ** | ** |
| K^+^ _(roots)_ (mmol g^-1^ DM) | ** | ** |
| Cl^-^ _(roots)_ (mmol g^-1^ DM) | ** | ** |
| Soluble carbohydrates _(leaves)_ (µmol g^-1^ DM) | ** | ** |
| Free amino acids _(leaves)_ (µmol g^-1^ DM) | ** | ** |
| Free proline _(leaves)_ (µmol g^-1^ DM) | ** | ** |
| Soluble proteins _(leaves)_ (mg g^-1^ DM) | ** | ** |
| Soluble carbohydrates _(roots)_ (µmol g^-1^ DM) | ** | ** |
| Free amino acids _(roots)_ (µmol g^-1^ DM) | ** | ** |
| Free proline _(roots)_ (µmol g^-1^ DM) | ** | ** |
| Soluble proteins _(roots)_ (mg g^-1^ DM) | ** | ** |
| APX _(leaves)_ (µmol H_2_O_2_ min.^-1^ g^-1^ DM) | ** | ** |
| CAT _(leaves)_ (µmol H_2_O_2_ min.^-1^ g^-1^ DM) | ** | ** |
| SOD _(leaves)_ (UA min.^-1^ g^-1^ DM) | ** | ** |
| LP _(leaves)_ (µmol MDA g^-1^ DM) | ** | ** |
| APX _(roots)_ (µmol H_2_O_2_ min.^-1^ g^-1^ DM) | ** | ** |
| CAT _(roots)_ (µmol H_2_O_2_ min.^-1^ g^-1^ DM) | ** | ** |
| SOD _(roots)_ (UA min.^-1^ g^-1^ DM) | ** | ** |
| LP _(roots)_ (µmol MDA g^-1^ DM) | ** | ** |

*; ** Significant at p ≤ 0,05 and p ≤ 0,01, respectively; ns, not significant.

ShDM (shoot dry mass), P_N_ (net CO_2_ assimilation rate), E (transpiration rate), gs (stomatal conductance), Chl *a* (chlorophyll *a* content), Chl *b* (chlorophyll *b* content), Chl *a* + *b* (chlorophylls *a* + *b* content), Car (carotenoids content),WRC (water relative content), EL (electrolyte leakage), WSD (water saturation deficit), WCS (water content at saturation), SUC (leaf succulence), SI (sclerophylly index), Na^+^ _(leaves)_, K^+^ _(leaves)_ and Cl^-^ _(leaves)_ (Na^+^, K^+^ and Cl^-^ contents in leaves, respectively), Na^+^ _(roots)_, K^+^ _(roots)_ and Cl^-^ _(roots)_ (Na^+^, K^+^ and Cl^-^ contents in roots, respectively), APX _(leaves)_, CAT _(leaves)_, SOD _(leaves)_, LP _(leaves)_ (ascorbate peroxidase, catalase, superoxide dismutase activities, and lipids peroxidation in leaves, respectively), APX _(roots)_, CAT _(roots)_, SOD _(roots)_, LP _(roots)_ (ascorbate peroxidase, catalase, superoxide dismutase activities, and lipids peroxidation in roots, respectively).
